# Supplementary material for: Achieving Cycling Stability in Anode of Lithium-Ion Batteries with Silicon-Embedded Titanium Oxynitride Microsphere
Source: Nanomaterials (Basel). 2022 Dec 27;13(1):132. doi: 10.3390/nano13010132 (PMC9823697; doi:10.3390/nano13010132)
Supplement: Supplementary file 1 [file nanomaterials-13-00132-s001.zip › nanomaterials-2117769-supplementary.pdf]

Supplementary material

Achieving Cycling Stability in Anode of Lithium-Ion Batteries with Silicon-Embedded Titanium Oxynitride Microsphere

Sung Eun Wang [1,†], DoHoon Kim [1,2,†], Min Ji Kim [1,3], Jung Hyun Kim [1], Yun Chan Kang [3], Kwang Chul Roh [1], Junghyun Choi [1], Hyung Woo Lee [2,4,*] and Dae Soo Jung [1,*]

1 Energy Storage Materials Center, Korea Institute of Ceramic Engineering & Technology (KICET), Jinju-si, 52851, Republic of Korea
2 Department of Nanoenergy Engineering, Pusan National University, Pusan, 46241, Republic of Korea
3 Department of Materials Science and Engineering, Korea University, Seoul 02841, Republic of Korea
4 Department of Nano Fusion Engineering and Research Center of Energy Convergence Technology, Pusan University, Pusan, 46241, Republic of Korea
* Correspondence: dsjung@kicet.re.kr (D.S.J.); lhw2010@pusan.ac.kr (H.W.L.)
† These two authors contributed equally to this work.

Table S1. Electrochemical performances of various Si composites applied as anodes for LIBs reported in the previous works.

Electrode material	Preparation method	Voltage window (V vs Li/Li^+)	Initial discharge/charge capacities (mA h g^{-1})	Reversible capacity (mA h g^{-1})	Rate capability (mA h g^{-1})	Ref.
Porous Si microsphere@C	Hydrothermal reaction	0.01-1.5	2367/1652 [0.2 A g^{-1}]	1665 (100) [0.2 A g^{-1}]	499 [4 A g^{-1}]	[16]
SiO_x-TiO_2@C	Sol-gel	0.01-2.5	1618/1011 [0.1A g^{-1}]	900 (200) [1 A g^{-1}]	542 [3.2 A g^{-1}]	[20]
Nitrogen-rich carbon/Si	Hydrothermal reaction	0.05-2.0	2394/1712 [0.1 A g^{-1}]	950 (100) [0.1 A g^{-1}]	579 [2 A g^{-1}]	[49]
Si@graphitized carbon	Magnesiothermic co-reduction	0.005-1.5	1306/534 [0.1 A g^{-1}]	681 (100) [0.2 A g^{-1}]	294 [2 A g^{-1}]	[50]
Al_2O_3 coated G/Si	Sol-gel	0.01-1.5	747/659 [0.05 C]	530 (100) [0.1 C]	-	[51]
Si-TiON (1:1)	Spray drying	0.01-1.5	2076/1663 [0.1 A g^{-1}]	1140 (100) [1 A g^{-1}]	603 [5 A g^{-1}]	This work

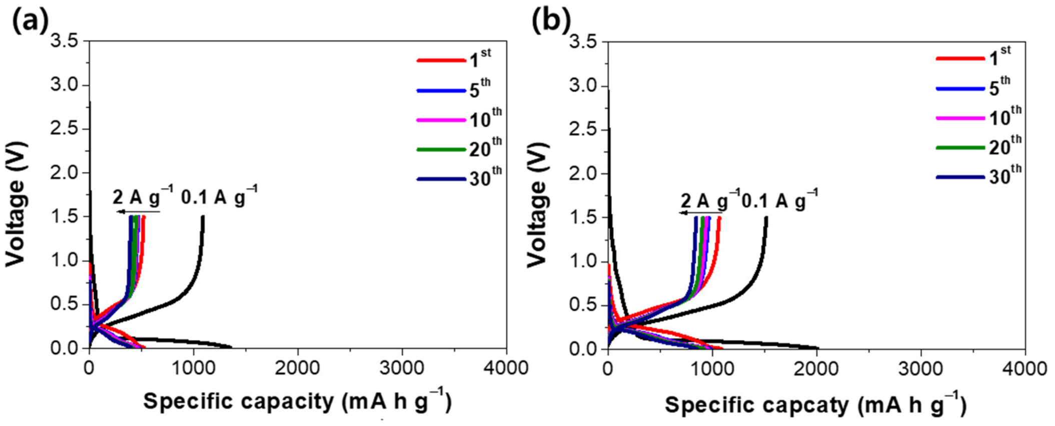

Figure S1. Voltage profiles for first and selected subsequent cycles at 0.1 A g^{-1} and 2 A g^{-1}, respectively: (a) Si-TiON (1:2) and (b) Si-TiO_2 (1:1).

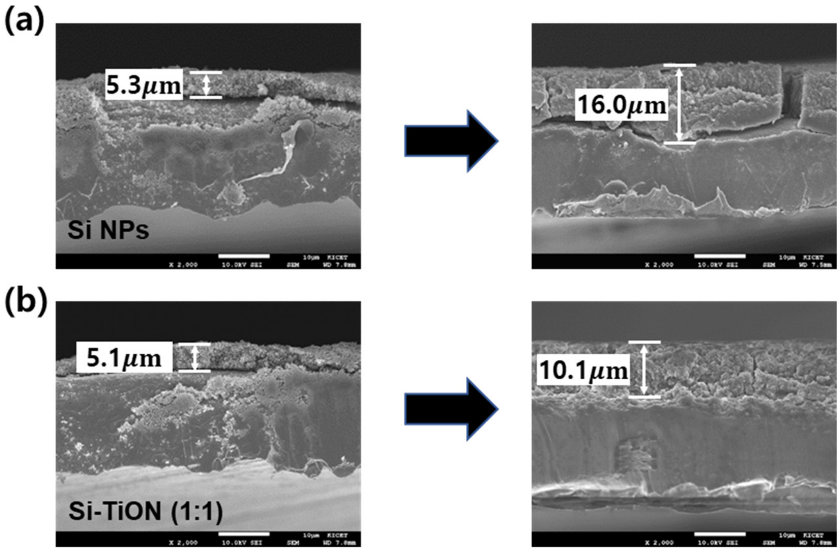

Figure S2. Cross-sectional FE-SEM images of (a) Si NPs and (b) Si-TiON (1:1) electrodes before and after 30 cycles at 2 A g^{-1}.

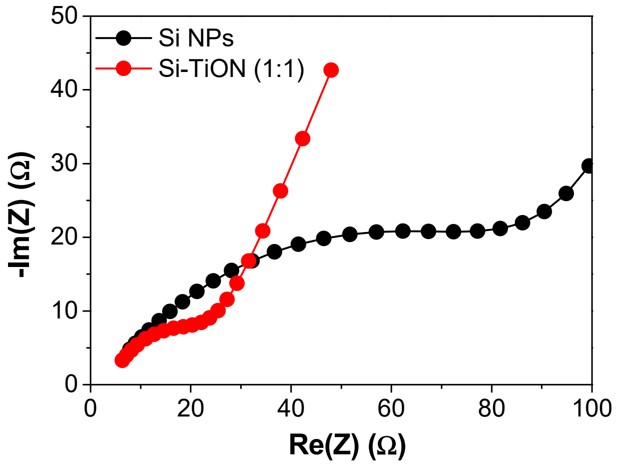

Figure S3. EIS profiles of Si NPs and Si-TiON (1:1) electrodes after 100 cycles at 1 A g^{-1}.
